# The Synergistic Toughening and Strengthening Effects of Cork Particles and Nanocellulose on Rosin-Based Epoxy Resin

**DOI:** 10.3390/polym14235064

**Published:** 2022-11-22

**Authors:** Jingrong Sun, Jinmeng Bai, Jingjing Li

**Affiliations:** College of Forestry, Northwest A&F University, Yangling, Xianyang 712100, China

**Keywords:** rosin-based epoxy resin, cork, nanocellulose, toughening, strengthening

## Abstract

In order to develop a bio-based epoxy resin with high mechanical and thermal performance, cork particles and nanocellulose were introduced into the rosin-based epoxy resin to improve the toughness, stiffness and thermal stability. The flexural properties of the epoxy composites indicated that the strength and modulus were reduced when the content of cork particles was relatively high (>3%) due to the low stiffness and modulus of cork itself. However, the flexural performance was significantly improved after the addition of 1% nanocellulose. In contrast to the flexural properties, the impact toughness results showed the synergistic toughening effects of nanocellulose and cork particles on the rosin-based epoxy resin. The highest impact toughness of 13.35 KJ/m^2^ was found in the epoxy composite with 1% cellulose nanofibers and 3% cork particles, an increase of 149.07% compared to the neat epoxy. Cork particle size also had a significant effect on the mechanical properties of the composites. Both the flexural and impact results showed first a rise and then a fall with a decrease in the cork size. TGA results indicated cork particles and nanocellulose could have a synergistic enhancing effect on the thermal stability of the rosin-based epoxy resin. This work can add value to rosin and cork waste and widen the industrial applications of the epoxy resin.

## 1. Introduction

In order to reduce crude oil consumption and the corresponding environmental impact, the use of sustainable resources to develop biopolymers has recently attracted much attention. Epoxy resin is the most widely used thermosetting resin at present, and has been widely used in the fields of coatings, encapsulation, composites and electronic applications [1,2,3,4,5,6]. Recently, some bio-based epoxy resin materials extracted from vegetable oils [7], cardanol [8], lignin [9], tannin [10], isosorbide [11] and rosin [12], have been reported. Rosin, which is obtained by heating the fresh resin extracted from pines, is a mixture of 40–60% isomeric abietic-type acids and 9–27% pimaric-type acids with respect to total rosin weight [13]. Hence, rosin-based epoxy resins can be synthesized with the rosin acid derivatives as raw materials due to the large hydrogenated three phenanthrene ring-containing skeleton in the structure. It was reported that the cured rosin-based epoxy resin displayed higher glass transition temperature, mechanical strength and modulus than the petroleum-based epoxy resin [14]. However, some problems with the rosin-based epoxy resin, such as high brittleness because of its high crosslinking density and poor fire-retarding qualities, limit its application in many fields [15]. Therefore, it is necessary to modify the rosin-based epoxy resin to improve its toughness, while retaining its excellent properties as an epoxy resin [16].

Cork, which is a part of the bark obtained by cutting broad-leaved *Quercus surber* or *Quercus variabilis*, is a natural and closed-cell biological material with a set of excellent properties, such as light texture, elasticity, admirable insulation and flame retardancy [17,18]. Some remarkable properties of cork-based composites, such as the high damage tolerance to impact loads, good thermal and acoustic insulation capacities and excellent damping characteristics, were obtained after cork was introduced into polymer matrices [19]. Hence, the development of cork-based composites is one of the most promising fields of cork technology since it is an effective way to increase recycling of cork processing residues and cork products [20]. Many studies have been conducted on the combination of cork and cork by-products with polymer matrices such as polyethylene [21], polypropylene [22], epoxy resin [23], poly(lactic acid) [24] and polycaprolactone [19]. In our previous study, cork was introduced into the rosin-based epoxy resin to improve the impact strength and thermal stability [25]. Analysis of the results of the mechanical properties indicated that the impact toughness of an epoxy composite with 3 wt% cork particles (100–200 mesh) was increased by 119% compared to the pure resin polymer. However, the modulus and rigidity of the epoxy resin was deteriorated when the amount of cork particles was high, due to the low stiffness of the cork itself. Therefore, how to obtain good impact toughness without decreasing the mechanical strength and stiffness is a difficult problem in the study of epoxy resin toughening.

Nanocellulose, as a kind of cellulose-based nanomaterial, exhibits a better enhancement effect than traditional cellulose fibers due to the characteristics of nanomaterials, such as nano-scale effect, high surface area, outstanding mechanical strength and high thermal stability, etc. [26,27,28]. Therefore, it was selected as a reinforcement material to increase the strength and rigidity of the epoxy resin in this study. Nanocelluloses can be divided into cellulose nanofiber (CNF) and nanocrystal cellulose (NCC) according to their morphology. CNF has highly ordered crystalline regions alternating with disordered amorphous domains [29]. NCC, which has rod-like nanoparticles and presents only crystalline regions, is mainly prepared by the acid hydrolysis method [30]. Compared to NCC, CNF presents a higher aspect ratio and a web-like structure due to the alternately arranged crystalline and amorphous structures [31]. Some research has focused on using nanocellulose as the reinforcement to improve the mechanical and thermal performance of epoxy resins by the impregnation method or solution blending method [32,33,34].

In this paper, we propose combining the strengthening and toughening properties of the rosin-based epoxy resin with nanocellulose and cork particles to develop a new type of biocomposite with high mechanical performance and thermal properties. The high elasticity of cork particles can increase the impact toughness of the rosin-based epoxy resin, and the rigid network structure of nanocellulose can improve the stiffness. The synergistic enhancement effect of cork particles and nanocellulose was analyzed by investigating the flexural performance, impact toughness, micro morphology and thermal stability of the epoxy resin composites. The results will provide a theoretical basis for broadening the application field of epoxy resin composites and improving the added values of rosin and cork waste.

## 2. Materials and Methods

### 2.1. Materials

*Quercus variabilis* cork was crushed into particles of different size by a plant crusher. Rosin-based epoxy resin was synthesized using gum rosin as raw material. Cellulose nanofiber (CNF) and nanocrystal cellulose (NCC) were prepared using Chinese white poplar flour as the raw material. All chemicals were of analytical grade and used without any further purification. The qualitative characteristics of all components are presented in Table 1.

### 2.2. Preparation of Composites

#### 2.2.1. Preparation of Rosin-Based Epoxy Resin

An amount of 250 g rosin and 1.25 g hydroquinone were added to a four-necked round-bottom flask equipped with a thermometer, an electric stirrer, a condensing reflux tube and a dropping funnel at constant pressure. Nitrogen was slowly added into the flask and the reaction system was heated. When heated to 220 °C, 45 mL acrylic acid was added into the flask dropwise through the feeding funnel over half an hour, and the crude acrylopimaric acid could be obtained by resting the whole system for 4 h, then cooling to 170 °C. The collected acrylopimaric acid was introduced into the sodium hydroxide ethanol solution at a concentration of 10% (*w*/*w*) to be purified. The system was heated to 60 °C and continuously stirred for 15 min. Finally, the sample was filtered and washed with distilled water to neutralize it, and dried for 24 h to obtain white powdered acrylopimaric acid.

An amount of 25 g of the above purified acrylopimaric acid and 75 g epichlorohydrin were added into a three-necked round-bottom flask with a thermometer, a magnetic stirrer and a condensation reflux tube. Then 0.65 g benzyl triethylammonium chloride was added into the system when the acrylopimaric acid was fully dissolved with an increase in temperature. The reaction mixture was stirred continuously for 90 min at 100 °C. When the acid value of the solution changed by less than 1 mg / g, the reaction was ended, and the esterification of acrylopimaric acid was obtained. Then, 10 g NaOH was added into the system at 70 °C for 3 h until the pH value did not change. After the reaction, the filtered sample was washed by distilled water to neutralize it. An amount of 250 mL petroleum ether was added into the sample for elution 1~2 times, and excess solvent was removed by a rotary evaporator. Finally, a light yellow transparent viscous liquid, namely the synthetic rosin-based epoxy resin, was obtained.

#### 2.2.2. Preparation of Nanocellulose

The preparation of CNF involved two steps, namely chemical treatment and mechanical treatment. The chemical treatment of poplar flour was conducted according to the well-known method to remove the hemicellulose and lignin [35]. The mechanical treatment was performed in a grinder (MKCA6-2, Masuko Sangyo Co. Ltd., Yamanashi, Japan) with a pair of stone disks to break up the fiber bundles. A water slurry with 1 wt% cellulose nanofibers was passed through the grinder 20 times with the grinding stone at 1800 rpm.

The preparation of NCC was according to the sulfuric acid hydrolysis method. The amorphous regions of cellulose were disintegrated by introducing a negative charge to the nanoparticles surface. The above CNF water slurry was hydrolyzed by 64% sulfuric acid at 45 °C for 45 min via continuous stirring in an ultrasonic bath at 20 kHz. When the color of suspension turned to dark yellow, distilled water was added immediately to stop the hydrolysis reaction. The suspension was then centrifuged and washed with distilled water to neutralize it. Finally, the colloidal suspension was sonicated for 10 min to homogenize the NCC sample.

#### 2.2.3. Preparation of Cork/Rosin-Based Epoxy Resin Binary Composites

*Quercus variabilis* cork was crushed into particles by a plant crusher and then sieved by a mesh-screen. The mesh-screen scales were <20 mesh, 20–40 mesh, 40–100 mesh, 100–200 mesh and 200–300 mesh. The above rosin-based epoxy resin was dissolved in ethanol solution and heated to 50 °C to reduce its viscosity. The curing agent, methyl hexahydrophthalic anhydride, was added to the rosin-based epoxy resin with a mass ratio of 1:1. Then, the cork particles were compounded with the rosin-based epoxy resin and curing agent system. The cork particle content was fixed at 1%, 2%, 3%, 4%, and 5 wt% based on the weight of the pure rosin-based epoxy resin polymer. The mixture was stirred under vacuum for 15 min at 70 °C to remove the ethanol and then transferred into a polytetrafluoroethylene (PTFE) mold. Finally, the samples were placed in an oven at 160 °C for 2 h for the epoxy curing, then the temperature was increased to 180 °C for 1 h. When the epoxy resin was fully cured, the composites were de-molded and used for characterization.

#### 2.2.4. Preparation of Nanocellulose/Rosin-Based Epoxy Resin Binary Composites

The as-prepared CNF and NCC were solvent-exchanged to anhydrous ethanol by successive centrifugation, and the sediments were fully dispersed by sonication to form CNF and NCC ethanol suspensions. The rosin-based epoxy resin was heated to 50 °C to reduce its viscosity, mixed with the CNF or NCC suspension, and stirred to form a uniform mixture. Then the same content of curing agent, methyl hexahydrophthalic anhydride, as the pure resin polymer was added to the mixture, followed by stirring for 30 min under ultrasonic conditions. The content of CNF or NCC was fixed at 0.2%, 0.5%, 1.0% and 1.5% of rosin-based epoxy resin. The mixture was stirred under vacuum for 15 min at 70 °C to remove the ethanol solvent, then poured into the PTFE mold and placed in an oven for the curing process. The temperature was set at 160 °C for 2 h, then increased to 180 °C for 1 h. When the epoxy resin was fully cured, the composites were de-molded and used for characterization.

#### 2.2.5. Preparation of Cork/Nanocellulose/Rosin-Based Epoxy Resin Ternary Composites

The ethanol solvent-exchanged CNF or CNC suspension was mixed with the rosin-based epoxy resin and curing agent and sonicated for 30 min to form a uniform mixture. The content of CNF or CNC was fixed at 1% of the pure epoxy resin polymer. Then, cork particles with different contents were added to the mixture and continuously stirred under vacuum at 70 °C for 15 min to evaporate the solvent. The cork/nanocellulose/epoxy resin ternary composite was obtained after it was poured into a PTFE mold and placed in the oven for curing. The temperature was set at 160 °C for 2 h, then increased to 180 °C for 1 h. When the epoxy resin was fully cured, the composites were de-molded and used for characterization.

### 2.3. Property Evaluation

#### 2.3.1. The Rosin-Based Epoxy Resin

##### Fourier Transform Infrared Spectroscopy (FTIR)

The FTIR spectra of the synthesized rosin-based epoxy resin before and after curing were recorded on a FTIR instrument (Nicolet IS10, Thermo Fisher Scientific, Walthman, MA, USA) equipped with a Germanium Attenuated Total Reflectance (ATR) in the wavenumber range of 400–4000 cm^−1^ and a resolution of 8 cm^−1^.

#### 2.3.2. The Nanocellulose

##### Field-Emission Scanning Electron Microscopy (FE-SEM)

The as-prepared CNF/NCC water suspension was freeze dried until the moisture content of the mixture became stable. The freeze-dried nanofibers were observed using an FE-SEM (HITACHI S-4800, Hitachi Limited, Tokyo, Japan). The samples were sputter-coated with gold for 30–60 s to avoid charging. The acceleration voltage was 3 kV and the coating current was 10 mA.

##### Transmission Electron Microscope (TEM)

The TEM observation was conducted on a Hitachi-H7650 (Hitachi, Ltd., Tokyo, Japan) at an acceleration voltage of 100 kV. A droplet of CNF/NCC suspension was deposited on a copper grid coated with a thin carbon film, followed by staining with phosphotungstic acid solution.

#### 2.3.3. The Cork Particles

##### Field-Emission Scanning Electron Microscopy (FE-SEM)

The pure cork particles with different sizes were observed using an FE-SEM (HITACHI S-4800, Hitachi Limited, Tokyo, Japan). The samples were sputter-coated with gold for 30–60 s to avoid charging. The acceleration voltage was 3 kV and the coating current was 10 mA.

#### 2.3.4. The Composites

##### Mechanical Properties

Pure resin polymer, cork/epoxy, nanocellulose/epoxy and cork/nanocellulose/epoxy composites were subjected to mechanical tests (flexural and impact). The flexural tests were carried out using a universal materials testing machine (AG-10TA, Shimadzu, Kyoto, Japan) according to ASTM D 638 with the crosshead speed of 1 mm/min and support span of 40 mm. Six defect-free specimens were selected for each test. The impact toughness was measured according to ASTM D 256 with an Izod impact test machine (QJBCX, Shanghai Qingji Instrumentation Technology Co., Shanghai, China) at room temperature. At least six specimens were tested for each measurement.

##### Field-Emission Scanning Electron Microscopy (FE-SEM)

The fracture surfaces of the cured composites were observed using an FE-SEM (HITACHI S-4800, Hitachi Limited, Tokyo, Japan). The samples were frozen in liquid nitrogen then quickly broken. Before observation, the surfaces of the sample was sprayed with gold for 30~60 s to reduce the discharge.

##### Thermogravimetric Analysis (TGA)

TGA was performed using an STA 449 F3 Jupiter (Jupiter, Netzsch, Freistaat Bayern, Germany) under nitrogen (60 mL min^−1^) from 30 to 600 °C at a scanning rate of 10 ℃ min^−1^. Pure cork, rosin-based epoxy resin, pure nanocellulose, cork/epoxy, nanocellulose/epoxy and cork/nanocellulose/epoxy composites were subjected to the TGA test.

## 3. Results and Discussion

### 3.1. Characterization of Rosin-Based Epoxy Resin

#### FTIR Analysis

**Figure 1:**
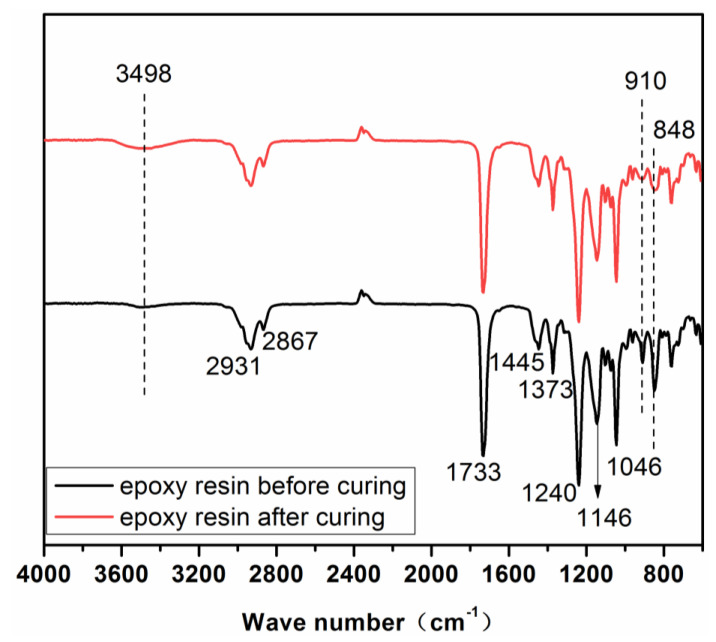
FTIR spectra of rosin−based epoxy resin before and after curing.

Figure 1 shows the FTIR spectra of the synthesized rosin-based epoxy resin before and after curing. For the epoxy resin before curing, the absorption peaks at 2931 cm^−1^ and 2867 cm^−1^ are attributed to the symmetric stretching vibration and antisymmetric stretching vibration of –CH_2_–, respectively. The peak at 1733 cm^−1^ belongs to the stretching vibration of C=O in the -COO– group, and the peaks at 1445 cm^−1^ and 1373 cm^−1^ belong to the bending vibration of -CH_3_ in the –CH (CH_3_)_2_^−^ group. The peak at 1240 cm^−1^ is due to the stretching vibration of C–O in the group –COOH, and the peak at 1146 cm^−1^ is due to the skeleton vibration of C–O–C. In addition, the peaks at 910 cm^−1^ and 848 cm^−1^ are also the characteristic peaks of the epoxy group. The absorption peaks in the infrared spectrum met the molecular structure and functional chemical groups of the epoxy resin, so it could be proved that the synthesized sample was the rosin-based epoxy resin. From the spectrum of the rosin-based epoxy resin after curing, it could be seen that the absorption peaks were almost the same as the epoxy before curing, except for the absorbance of peaks at 910 cm^−1^, 848 cm^−1^ and 3498 cm^−1^. The absorbance of peaks at 910 cm^−1^ and 848 cm^−1^ are much lower than those on the spectrum of the epoxy resin before curing, and these two peaks nearly disappeared. However, the absorbance of peak at 3498 cm^−1^ is higher than that of the epoxy before curing. These changes indicated the curing reaction of the rosin-based epoxy resin.

### 3.2. Characterization of Nanocellulose

#### Microscopic Morphology Observation

**Figure 2:**
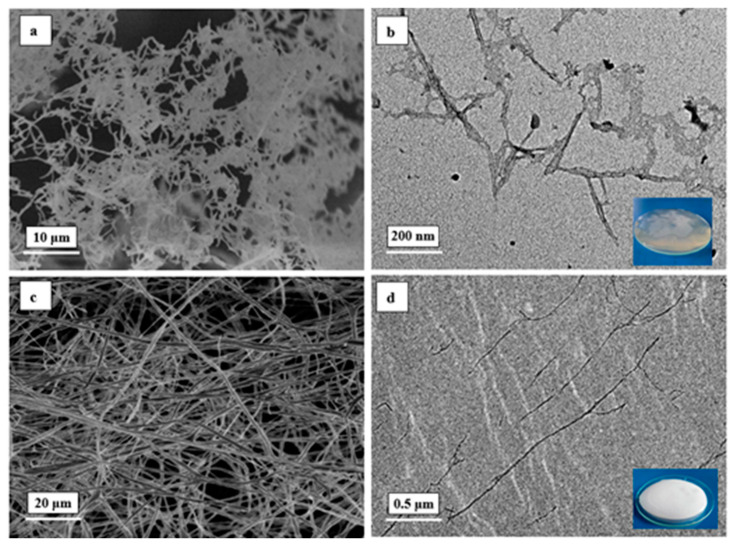
Microscopic morphology of nanocellulose: (**a**) FE-SEM image of NCC, (**b**) TEM image of NCC, (**c**) FE-SEM image of CNF and (**d**) TEM image of CNF.

The FE-SEM and TEM images of poplar NCC and CNF are shown in Figure 2. From Figure 2a,b, it could be seen that the short rod-like NCC exhibits disintegrated fine fibrous structure rather than large packed structure. The isolated NCC had an average diameter of 5–50 nm and length of 200–500 nm. It could also be observed that NCC was homogeneous and well-dispersed in the aqueous solution with good colloidal suspension stability. Compared to NCC, CNF had a classical web-like network structure with long entangled cellulosic filament. The fibers in Figure 2c,d were highly uniform even over an extensive area, with an average diameter of 50–100 nm and length exceeding 1 μm. Hence, the aspect ratio of poplar CNFs was up to 500–2000. It indicates that CNF with a larger aspect ratio is associated with better reinforcing effect than NCC in improving the mechanical properties of the polymeric matrix [32,35].

### 3.3. Characterization of Cork Particles

#### Microscopic Morphology Observation

**Figure 3:**
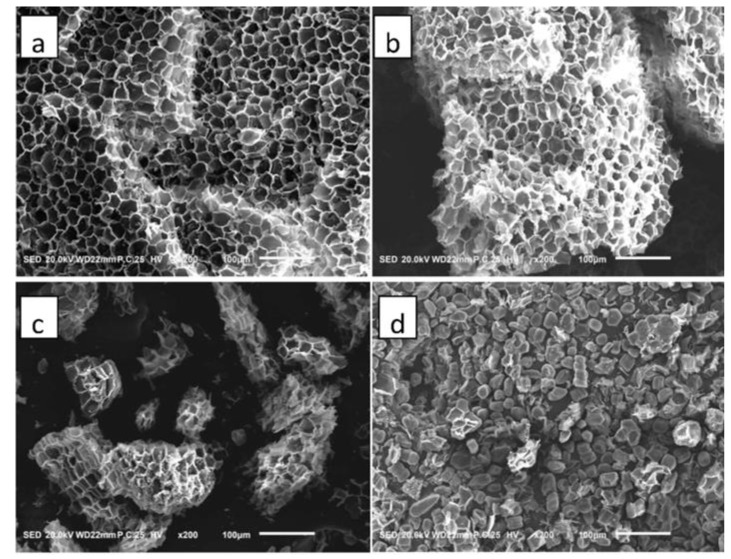
FE-SEM images of cork particles with different sizes: (**a**) <20 mesh, (**b**) 40–100 mesh, (**c**) 100–200 mesh, (**d**) 200–300 mesh.

The microstructure of the pure cork particles with different sizes is revealed in Figure 3. It could be seen that the structure of cork cells was honeycomb-like, and most cells were thin-walled cells. The arrangement of cork cells in the three sections was different; being mainly honeycomb hexagon on the tangential section but brick quadrilateral on the transverse and radial sections. When the size of cork particles was <20 mesh (Figure 3a), cork presented a honeycomb-like structure composed of intact cells with no empty spaces; therefore, these cells were closed units filled with air. When the size of cork particles decreased to 40–100 mesh (Figure 3b), some damaged and open cells resulting from the grinding process could be observed clearly. With the further reduction in cork particle size to 100–200 mesh (Figure 3c), more cork cells were damaged, and more cell wall fragments were found. When the size decreased to 200–300 mesh (Figure 3d), there was hardly any intact cork cells but there was fragments of cell walls present. The elastic performance of cork is mainly decided by the amount of intact closed cells, therefore the increase in the damaged cells can have a negative effect on improving the impact toughness of the epoxy resin matrix.

### 3.4. Characterization of Composite Materials

#### 3.4.1. Mechanical Properties

##### Flexural Performance

**Figure 4:**
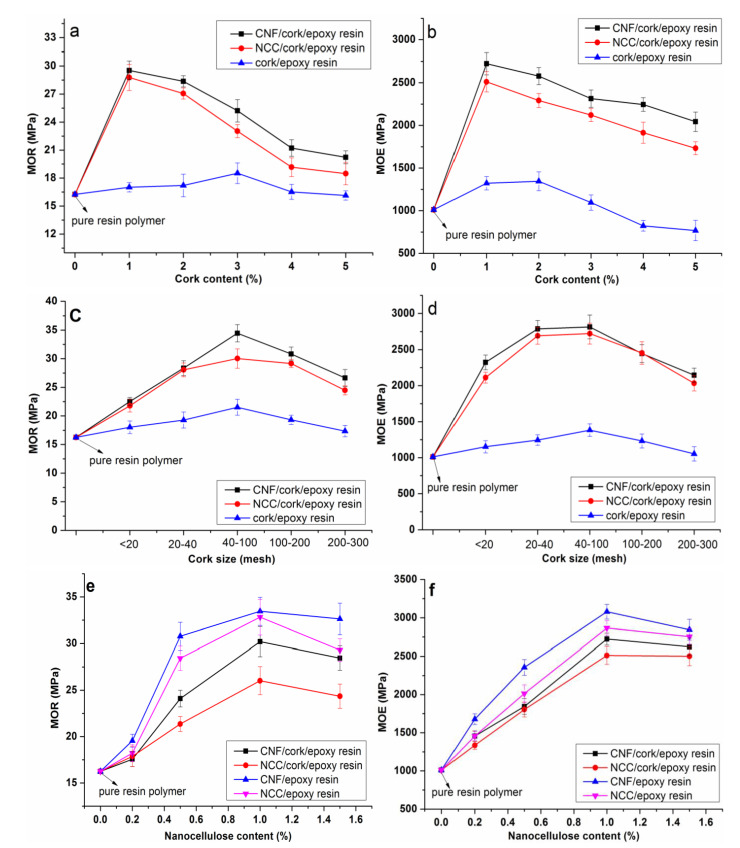
The flexural strength and modulus of the rosin−based composites, (**a**,**b**) MOR and MOE of composites with different cork contents, (**c**,**d**) MOR and MOE of composites with different cork sizes, (**e**,**f**) MOR and MOE of composites with different nanocellulose contents.

The flexural strengths and moduli of the cork/nanocellulose/epoxy resin composites are presented in Figure 4. As can be seen from Figure 4a,b, an effect of the cork particle content on the modulus of rupture (MOR) and modulus of elasticity (MOE) of the composites with 1% nanocellulose could be observed. For the cork/epoxy binary composite, when the cork content was less than 3%, the addition of cork did not affect significantly the flexural strength and modulus of the composite. However, the MOR and MOE were obviously reduced when the cork content was more than 3%. The decrease in flexural performance can be attributed to the characteristic lower stiffness and foamed structure of cork particles, which make the composites much more ductile [23]. Compared to the cork/epoxy binary composite, the flexural strength and modulus of the cork/CNF (NCC)/epoxy ternary composites increased remarkably due to the introduction of nanocellulose. The MOR and MOE of the pure epoxy resin polymer were 16.25 MPa and 1011 MPa, respectively, and they were 17.02 MPa and 1322 MPa in the cork/epoxy binary composite with 1% cork particles, respectively, while the corresponding result increased to 29.53 MPa and 2723.45 MPa, respectively, when 1% CNF was added into the composite system. The improvement in the bending performance is ascribed to the formation of a rigid nanofiber network, which maximizes stress transfer across the interface to promote the mechanical strength [36]. Additionally, it should be noted that although the MOR and MOE of the nanocellulose/cork/epoxy ternary composite decreased with added cork when the content was >1%, the corresponding values were still much higher than those of the cork/epoxy binary composites without nanocellulose.

The effects of cork particle size on the flexural performance of the composites are presented in Figure 4c,d, where the content of cork is fixed at 3 wt%. The MOR and MOE of all the composites showed a trend of first rising and then falling with a decrease in cork particle size. The maximum values were obtained in the composites with the cork particle size of 40–100 mesh. Compared to the larger size of cork particles (<40 mesh), the smaller size of cork particle represents a larger surface area and more opportunities to interact with the epoxy resin matrix, and this can lead to much stronger interaction, blocking the movement of matrix molecular chains and having a positive effect on the stress transfer in the interface [37]. Hence, the composite prepared with larger size of cork particles might produce more cavities on the interface resulting in the reduction in the mechanical properties [38]. However, when the size of cork particles decreased to 200–300 mesh, the agglomeration effect of the cork particles in the matrix was intensified and the dispersion uniformity worsened, leading to a decrease in the flexural strength and modulus. In addition, it was reported that for the cork composites, cork particles should have a considerable number of intact cells, so that they can work together as a whole to cause the toughening effect of cork particles [21]. With the size of cork particle decreasing, the collapse of the cellular structure of cork would increase accordingly. The destruction of cork cells was also responsible for the decline of MOR and MOE for the composites.

Similarly, the effects of nanocellulose content on the flexural strength and modulus of composites are indicated in Figure 4e,f. When the nanocellulose content was <1%, the MOR and MOE increased remarkably with an increase in nanofiber content. However, a slight decrease in MOR and MOE could be observed when the nanofiber content was more than 1%. The results indicated that a relatively high content of nanocellulose had a negative effect on the mechanical performance of the composites, since the interfacial compatibility between the nanocellulose and epoxy resin matrix became poor due to the non-uniform dispersion of a high content of nanofiber in the matrix. Compared to NCC, a better reinforcing effect of CNF on the epoxy resin could be observed. The reason could be inferred as follows, CNF has a much more refined structure, higher aspect ratio and larger specific surface area than NCC particles; thus, there will be much more nano–micro scale fibers absorbing the epoxy resin molecular chains simultaneously, leading to an increase in the mechanical properties [39]. Similar to Figure 4a,b, the MOR and MOE of the ternary composites with nanocellulose were much higher than those of the cork/epoxy binary composite. The significant enhancement effect of nanocellulose could be due to the solid embedding of nanofibers into the epoxy resin, generating a homogenous structure of the nano-filled matrix.

##### Impact Toughness

**Figure 5:**
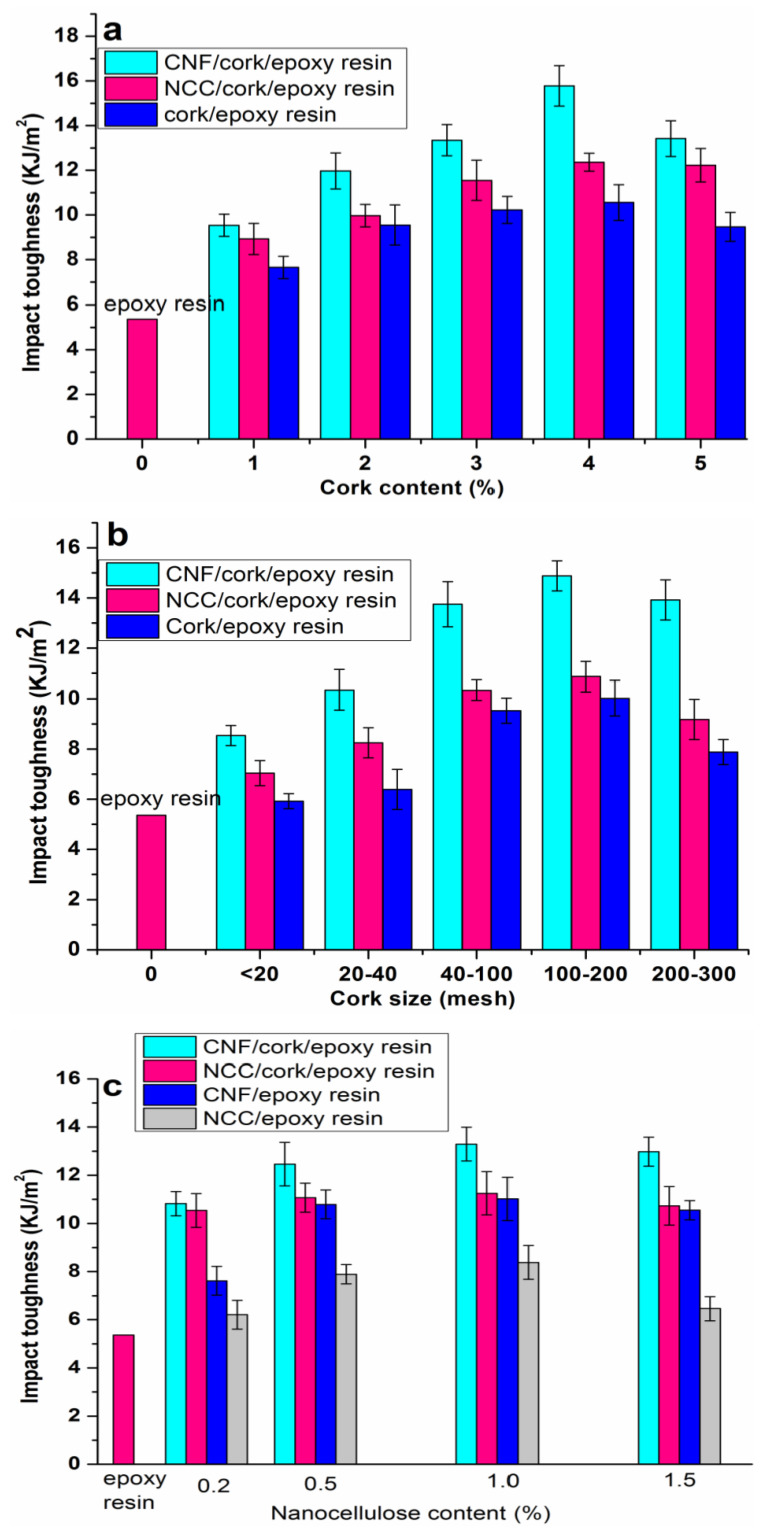
The impact toughness of the rosin-based composites: (**a**) composites with different cork contents, (**b**) composites with different cork sizes, (**c**) composites with different nanocellulose contents.

Figure 5 shows the impact toughness of the nanocellulose/cork/rosin-based epoxy resin composites. The effect of cork content on the impact toughness of the composites with 1% nanocellulose can be observed in Figure 5a. The impact toughness of pure rosin-based epoxy resin polymer was 5.36 KJ/m^2^. When adding 1% cork particles, the impact toughness of the composite was increased to 7.65 KJ/m^2^, an increase of 42.72% compared to the resin matrix, indicating that the addition of cork particles significantly improved the toughness of rosin-based epoxy resin. When the content of cork particles was 2%, 3%, 4% and 5%, the impact toughness of the composites was 9.55 KJ/m^2^, 10.23 KJ/m^2^, 10.56 KJ/m^2^ and 9.47 KJ/m^2^, respectively. In contrast to the flexural properties, the impact toughness of the composites showed an obvious upward trend with an increase in cork particles when the content was less than 4%. The toughening effect of the cork particles can be attributed to their closely arranged cell structure. Cork exhibits an alveolar structure without empty spaces between contiguous cells; therefore, these cells are closed units and the interior is full of air [23]. The closed characteristics of cork cells contribute to the excellent resilience and flexibility [25]. When the cork material is damaged by the impact load, the energy in the surrounding environment can be strongly absorbed by the highly elastic cork cells. Moreover, it could be observed that the addition of nanocellulose into the cork/epoxy resin system further improved the impact toughness of the rosin-based epoxy resin. Both the CNF/cork/epoxy and NCC/cork/epoxy ternary composites exhibited higher impact resistance compared to the cork/epoxy binary composite without nanocellulose. The results indicated the synergistic toughening effect of nanocellulose and cork particles on the rosin-based epoxy resin.

The effect of cork particle size on the impact toughness of the composites with 3% cork is shown in Figure 5b. The impact toughness increased significantly with the decrease in cork particle size when the size was below 200 mesh. Hence, the highest impact toughness was obtained in the CNF/cork/epoxy ternary composite with a cork size of 100–200 mesh, measured to be 14.41 KJ/m^2^, an increase of 168.84% compared to the neat rosin-based epoxy resin. Within a certain range, cork particles of smaller size have larger specific surface area and contact area with the bonded epoxy matrix compared to cork particles of larger size. Therefore, smaller cork particles have better wettability to the epoxy resin, leading to a more effective transfer of the stress load from the matrix to the cork particles. However, with a further decrease in cork particle size (200–300 mesh), the impact toughness of the composite had a decline in the trend. This reduction can be ascribed to the damage to a large amount of cork cells. It was reported that cork cells had good impact behavior only when a group of intact cells were present [24]. During the milling process to prepare the cork particles, some intact cells were damaged on the walls, which could weaken the damping effect of the cork and reduce the energy absorption. Barbosa reported that the use of cork was different from the particles normally used as reinforcements, for example rubber particles. The cork size should be much larger, at least above 30 microns when included in the epoxy matrix. Below this value, the cork had no effect on increasing the toughness.

Figure 5c shows the effect of nanocellulose content on the impact toughness of the composites. It could be seen that with an increase of nanocellulose, the improvement of impact resistance was very significant when the nanocellulose content was less than 1%. In contrast to the flexural performance, the impact toughness of the CNF (NCC)/cork/epoxy ternary composites was much higher than that of the CNF (NCC)/epoxy binary composites without cork particles. This indicates that both cork particles and nanocellulose had positive effects on improving the impact resistance of the rosin-based epoxy resin. When the CNF content was fixed at 15 and the cork particle content was 35, the impact toughness of the composite reached its maximum, measured at 13.35 KJ/m^2^, an increase of 149.07% compared to the neat epoxy resin. It could also be observed that the impact toughness of the composites with CNF was significantly higher than that of the composites with NCC. This is because CNF has a higher aspect ratio and larger specific surface area than the NCC particles. The strong network structure formed by the long CNF can act as a crack stopper and suppress the crack propagation.

#### 3.4.2. FE-SEM

**Figure 6:**
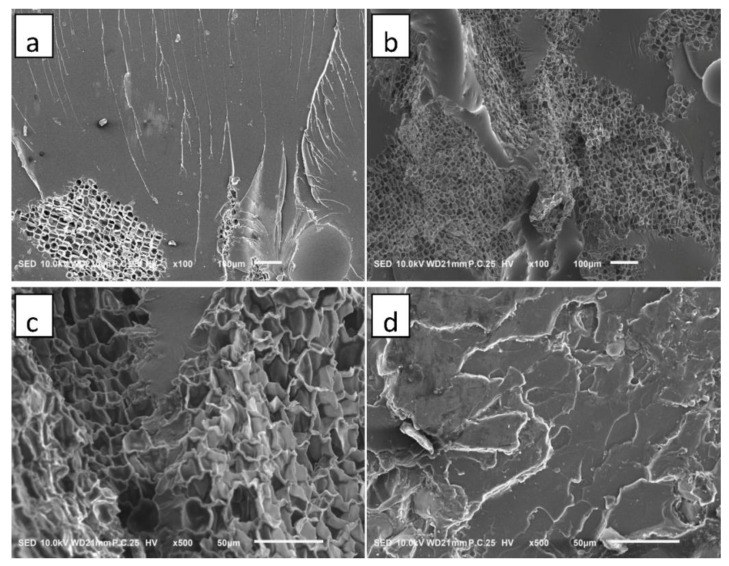
FE-SEM images of fracture surfaces for the cork/epoxy resin binary composites: (**a**) 1% cork, 40–100 mesh, (**b**) 5% cork, 40–100 mesh, (**c**) 5% cork, 40–100 mesh, (**d**) 5% cork, 200–300 mesh.

Figure 6 presents the FE-SEM images of the fractured surfaces for the cork/epoxy resin binary composites with different cork contents and cork particle sizes. At a relatively low content of cork particles (1%, Figure 6a), only a few cork cells in the epoxy resin could be observed. However, when the cork content increased to 5% (Figure 6b), much more intact cork cells were dispersed in the epoxy matrix. The FE-SEM images indicated that the peculiar morphology of the cork cells was, in general, maintained after the grinding and compounding processes. This was very important because many intrinsic properties of cork, such as the low density, elasticity and insulation properties, mainly arise from the peculiar morphology. In Figure 6a, multiple crack paths can be observed on the fractured surface of the resin matrix; therefore, the crack pinning or crack-front trapping was the main mechanism of cork particles toughening the epoxy resin [25]. Cork particles can induce the epoxy resin matrix to produce silver grain or shear band, which makes the matrix yield and absorb a lot of energy, therefore improving the impact resistance [39]. In addition, there were no obvious gaps or voids between cork particles and epoxy resins, indicating a good interfacial adhesion. The microstructure of cork/epoxy resin binary composites with different cork particle sizes is presented in Figure 6c,d. In the case of the composite with large cork size (40–100 mesh), the characteristic cellular structure of cork particles was still visible, but when the cork size decreased to 200–300 mesh, the morphology of cork cells has completely disappeared in the epoxy matrix. The damage of cork cells is related to the lower impact toughness of the composite with smaller cork particles. 

**Figure 7:**
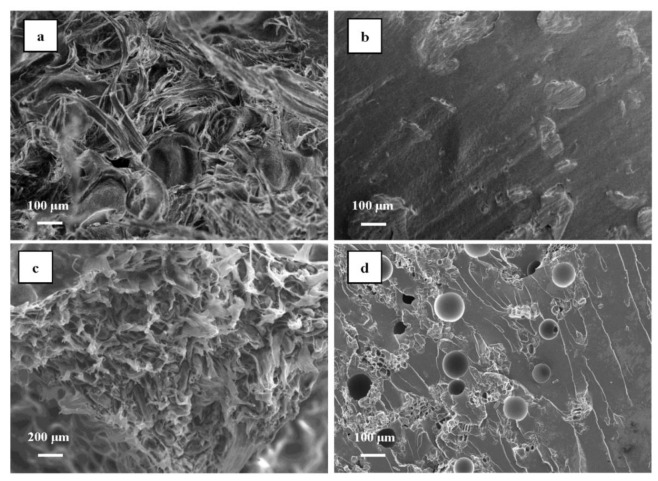
FE-SEM images of fracture surfaces for the nanocellulose/cork/epoxy resin composites: (**a**) CNF/epoxy, (**b**) NCC/epoxy, (**c**) CNF/cork/epoxy, (**d**) NCC/cork/epoxy.

Figure 7 shows the morphology of the fracture surface for the composites with nanocellulose. The fracture surfaces of the CNF (NCC)/epoxy resin binary composites without cork particles are presented in Figure 7a,b. From Figure 7a, it can be seen that the typical network structure of CNF still maintains the long and refined fiber appearance, making the fracture surface rather rough and uneven. Compared to CNF, the fracture surface of the NCC/epoxy resin composite (Figure 7b) was much smoother and flatter, suggesting a brittle fracture failure mode under the external impact load. CNF, with a high aspect ratio and large specific surface area, can not only deform itself, but can also produce some fine cracks around the impact area to restrict the free movement of polymer chains by the crack tip pinning, leading to higher impact toughness than NCC. The fracture surfaces of the CNF (NCC)/cork/epoxy ternary composites are presented in Figure 7c,d. Similar to the CNF/epoxy resin binary composite, the CNF/cork/epoxy resin ternary composite (Figure 7c) showed a much rougher fracture surface compared to the NCC/cork/epoxy resin composite (Figure 7d). In Figure 7c, the intact cork cells and CNF bundles can be observed clearly, contributing to a synergistic toughening effect on the rosin-based epoxy resin. However, in Figure 7d, many lines on the fracture surface of the NCC/cork/epoxy resin ternary composite can be seen. These rough lines indicate that the fracture failure of this composite was in a plastic yield fracture mode, which is beneficial in that it consumes more energy during fracture, so that the impact toughness of the composite could be improved [25]. Additionally, it was noted that the cork particles and nanocellulose were randomly distributed in the epoxy matrix, and the agglomerate of these fillers results in the stress concentration and thus affects the overall macro performance of the composite.

#### 3.4.3. Thermal Stability

**Figure 8:**
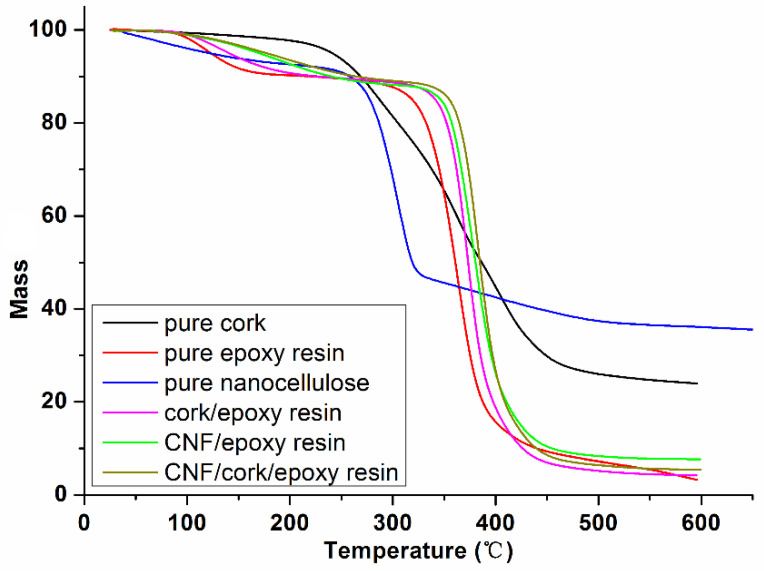
Thermogravimetric curves of the CNF/cork/epoxy resin composites.

The thermal stability of pure cork, pure rosin-based epoxy resin, pure nanocellulose, cork/epoxy, CNF/epoxy and CNF/cork/epoxy composites measured by TGA in the temperature range of 25–600 °C is presented in Figure 8. The degradation intensity of pure cork increased with temperature and heating time. Below 200 °C, the mass loss was relatively small. Substantial mass loss mainly occurred in the temperatures between 250 °C and 400 °C. At 400 °C, 60% of the cork mass was already lost. The chemical changes of cork during the thermo-decomposition process could be summarized as follows: Extractives were firstly volatilized (<100 °C), and then hemicellulose and cellulose were degraded (180–250 °C). Finally, lignin and suberin were decomposed (250–350 °C) [40]. For the rosin-based epoxy resin, the thermal stability was poorer than that of the cork materials. The temperature of 55 weight loss and the char yield for the rosin-based epoxy resin were 122.9 °C and 3.215, respectively, and the corresponding values were 240.8 °C and 23.945 for the pure cork particles. Hence, the introduction of cork particles could have the potential to improve the thermal stability of the rosin-based epoxy resin. The temperature of 55 weight loss and the char yield for the cork/epoxy resin binary composite were 140.8 °C and 4.725, respectively, which were higher than the values of the neat rosin-based epoxy resin. Similar to cork, the addition of nanocellulose into the polymer matrix could increase the thermal stability due to the extremely low thermal expansion coefficient of around 0.1 ppm/K for cellulose [41]. The temperature of 55 weight loss and the char yield for the CNF/epoxy resin binary composite were 170.2 °C and 7.625, respectively. It can be found that the effect of CNF on improving the thermal stability of the epoxy resin was more significant than that of the cork materials. For the CNF/cork/epoxy resin ternary composite, the temperature of 55 weight loss and the char yield were 178.1 °C and 5.395, respectively. These results indicated that the thermal stability of the ternary composite was better than that of the binary composite, and the addition of cork particles and nanocellulose could have a synergistic enhancing effect on the thermal stability of the pure rosin-based epoxy resin polymer.

## 4. Conclusions

In this work, the synergistic toughening and strengthening effects of cork particles and nanocellulose on the rosin-based epoxy resin were investigated in detail. The flexural results indicated that the MOR and MOE of the epoxy composites were obviously reduced when the cork content was more than 3% because of the low stiffness of cork itself. However, the flexural performance was improved significantly after the addition of 1% nanocellulose, suggesting a network structure-strengthening effect of nanocellulose. The impact toughness results showed the synergistic toughening effects of nanocellulose and cork particles on the rosin-based epoxy resin. The highest impact toughness of the composite with 1% CNF and 3% cork particles was 13.35 KJ/m^2^, an increase of 149.07% compared to the neat epoxy resin. Cork particle size also had an important effect on the mechanical properties of composite. The flexural and impact performance of the composites both exhibited a rise followed by a fall with a decrease in cork size. In addition, CNF exhibited a better reinforcing effect on the epoxy resin than NCC due to the higher aspect ratio. TGA results indicated that both the addition of cork particles and nanocellulose could improve the thermal stability of the rosin-based epoxy resin.

## Figures and Tables

**Table 1:** Characteristics of all components for composites.

Components	Source	Characteristics
Cork particles	Collected from Qinba Mountain in China, Crushed into particles with a crusher	The size of cork particles: >20 mesh, >830 μm; 20–40 mesh, 830–380 μm; 40–100 mesh, 380–150 μm; 100–200 mesh, 150–75 μm; 200–300 mesh, 75–48 μm
Rosin	Purchased from Wuzhou Pine Chemicals Ltd. (Suzhou, China)	WW grade; Color: light yellow; Softening point: ≥76 °C Acid value: ≥166 mg KOH/g
Nanocellulose	Using Chinese white poplar flour as raw materials	The size of poplar flour: 40–100 mesh, 380–150 μm
Chemicals	Purchased from Shanghai Aladdin Bio-Chem Technology Co. Ltd. (Shanghai, China)	Analytical grade

## Data Availability

All the data generated during this study are included in this article.
